# Medical Data Analysis Meets Artificial Intelligence (AI) and Internet of Medical Things (IoMT)

**DOI:** 10.3390/bioengineering10121370

**Published:** 2023-11-29

**Authors:** Manoj Diwakar, Prabhishek Singh, Vinayakumar Ravi

**Affiliations:** 1 Department of Computer Science and Engineering, Graphic Era Deemed to Be University, Dehradun 248002, India; 2 School of Computer Science Engineering and Technology, Bennett University, Greater Noida 201310, India; prabhisheksingh88@gmail.com; 3 Center for Artificial Intelligence, Prince Mohammad Bin Fahd University, Khobar 34754, Saudi Arabia; vravi@pmu.edu.sa

AI is a contemporary methodology rooted in the field of computer science. It involves the creation of programs and algorithms that fill devices with the ability to exhibit intelligence and effectiveness in carrying out tasks typically requiring human expertise. This is achieved through the utilization of various techniques, such as machine learning (ML), deep learning (DL), conventional neural networks (CNN), fuzzy logic, and speech recognition. At the same time, the IoMT arose as an innovative bio-analytical instrument that integrates interconnected biomedical equipment with a software application to enhance human well-being. The implementation of AL and IoMT on medical data improves the user’s quality of life, providing intelligent healthcare services. Technologies like AI and IoMT enhance the analysis and processing of medical data in various applications. The Special Issue will provide a concise summary of recent advancements in the subject, identify the existing knowledge gaps, elucidate how these gaps are being addressed, and culminate in a primary emphasis on future research that warrants attention.

The word “AI” was established by John McCarthy at a 1956 summit dedicated to exploration in this field. Nevertheless, Alan Turing, an influential figure in the field, introduced the concept of robots potentially emulating human behavior and exhibiting cognitive abilities. He devised the Turing test to distinguish between people and machines [1]. The use of AI methods has significant promise for integration across several domains within the realm of medicine. There is a need for further well-planned clinical studies prior to the use of these emerging strategies in the actual clinical environment [2]. Automated analytical systems have been widely recognized as database systems that exhibit the capability to scan medical images using computer technology and effectively handle large volumes of data [3].

The integration of health-specific indicators with an Internet of Things (IoT)-based health-observing system presents a complex study area that poses challenges in combining them with the capacity to handle massive amounts of data. Numerous concepts are posited to delineate the potential methodologies for monitoring and analyzing health issues by means of IoT-based medical big data using deep learning algorithms [4]. The substantial growth seen in the market for IoT medical devices may be attributed to the advancements in high-speed networking technologies, as well as the rising prevalence of wearable devices, smartphones, and other mobile platforms within the healthcare sector [5].

We started this Special Issue with the goal of promoting the use of AI and IoMT in the analysis and processing of medical data, and we ended up with six papers in total, including all the articles. These address several topics, including the prediction of COVID-19, the classification of endoscopic images, the classification of ECG heartbeats, the compression of bio-signals, and the evaluation of model correctness in eyes-open and eyes-closed EEG data. The section that follows provides an overview of the contributions.

The limitations of AI in the healthcare business are as follows: Ethical issues include a range of concerns pertaining to equitable treatment, responsible conduct, open disclosure, and the preservation of human dignity in the development and use of AI. Furthermore, they involve the safeguarding of privacy, the acquisition of informed consent, and the preservation of autonomy for both patients and healthcare professionals. Legal issues include the establishment and oversight of liability, accountability, and ownership pertaining to AI systems and their consequences. Additionally, adherence to both current and evolving laws and regulations is crucial [6]. Social issues include the examination and resolution of the possible implications of AI on human relationships, trust, communication, and empowerment within the healthcare sector. Additionally, this involves the effective management of the expectations, perceptions, and attitudes of many stakeholders. Technical challenges include the establishment of the credibility and dependability of AI systems and data, as well as the resolution of concerns related to security and compatibility. Additionally, it is crucial to address the constraints, prejudices, and uncertainties inherent in AI algorithms and models [7].

The application of IoMT in the healthcare industry is, essentially, the use of a groundbreaking technology, but it presents a series of obstacles that need resolution. One of the primary challenges is the matter of privacy and security [8]. The transmission of sensitive medical information across many devices and networks poses a potential vulnerability to cyber-attacks and data breaches. Another significant obstacle is the need for universally accepted and standardized procedures [8].

In [9], the authors propose a set of pre-trained algorithms that can correctly categorize endoscopic images of GI diseases and disorders. Classifying illnesses of the gastrointestinal system is the focus of this research, which proposes a weighted average ensemble model dubbed GIT-NET. In this review article [10], the authors set out to bring the reader up to speed on the latest research and applications of AI in perinatology, with a particular emphasis on newborn critical care and the possibilities presented by precision medicine. The research in [11] shows that IoT wearable devices with AI-based algorithms built into them were highly successful and efficient in detecting and anticipating insights related to COVID-19. The study’s findings show that AI is an exciting field of study with vast potential for improving healthcare worldwide in areas such as illness prediction, disease forecasting, medication discovery, and healthcare infrastructure development. 

According to the study in [12], data from electronic health records are shown as increasingly valuable assets for empirical research. Knowledge of the electronic health records system and its differences from existing observational data is necessary for the ML-based transformation of real-world data and electronic health records data for research and real-world evidence. The research in [13] questioned medical researchers at the University of Luebeck and the University Hospital of Tuebingen to ascertain their current levels of awareness of AI chatbots, specifically in the context of the healthcare environment. A quantitative survey and qualitative analysis of focus groups were used to learn how medical students felt about artificial intelligence and chatbots in the medical field. This would help determine what is needed to successfully include AI in future medical education. The goal was to obtain a general comprehension of the technology’s possible benefits, drawbacks, and dangers.

In [14], the authors suggested a compression technique for bio-signals well-suited to IoMT use. This approach uses block-based HWT to isolate features from the input signal and then uses the innovative COVIDOA to choose the most significant characteristics for reconstruction. Utilizing wireless communication transmission technologies from the industrial IoT, a smart data transmission model is suggested for use in the IoMT [15]. This model compensates for the flaws of previous IoMT models with its capacity for high-quality data transfer, accurate accident diagnostic categorization, and real-time anomaly monitoring. In [16], researchers created a new model for energy-conscious clustering and medical data categorization using the squirrel search algorithm and IoT. Maximum energy efficiency and accurate disease detection in an IoT setting are also goals of the proposed method. In [17], researchers employ a novel pre-trained DL model to provide an efficient measurement of prediction accuracy. To build accurate medical signs, treatments, and diagnoses, it is necessary to conduct a thorough analysis, such as self-supervised transfer learning, to monitor the effects of the DL models. 

There is a marked lack of quantitative research in favor of qualitative approaches in the bulk of the papers. Statistical modeling must be carried out to measure how each big data approach affects clinical routines [18]. Healthcare strategy, quality of service, resilience, and agility may all be improved and optimized with the use of AI and IoMT, giving researchers and practitioners more industrial control over the system. There is much room for growth in the field of human resource development due to the lack of studies on educating employees to adopt AI and IoMT [18]. Surgeons undertaking complex procedures may find the metaverse to be a helpful tool, ultimately leading to better patient care. New metaverse-based solutions may provide patients with immediate feedback on their test results. Since clinical studies are needed to establish whether or not the metaverse is a valuable tool for surgery, its implementation will occur gradually at first. Like robot-assisted surgery, we expect the metaverse to gain popularity as its applications expand [19].
